# Folate-mediated mitochondrial targeting with doxorubicin-polyrotaxane nanoparticles overcomes multidrug resistance

**DOI:** 10.18632/oncotarget.3090

**Published:** 2014-12-30

**Authors:** He Wang, Henghui Yin, Fengjiao Yan, Mingna Sun, Lingran Du, Wei Peng, Qiuli Li, Yinghong Feng, Yi Zhou

**Affiliations:** ^1^ Department of Oncology, The Second Affiliated Hospital of Guangzhou Medical University, Guangzhou, Guangdong, China; ^2^ The College of Pharmaceutics Science, Guangzhou Medical University, Guangzhou, Guangdong, China; ^3^ Center of Breast Disease, The First Affiliated Hospital of Sun Yat-Sen University, Guangzhou, Guangdong, China; ^4^ Department of Otorhinolaryngology, The First Affiliated Hospital of Sun Yat-Sen University, Guangzhou, Guangdong, China; ^5^ Department of Pharmacology, Uniformed Services University of the Health Sciences, Bethesda, Maryland, USA

**Keywords:** Polyrotaxanes, Functional DOX nanoparticles, Multidrug resistance, Mitochondrial signaling pathway

## Abstract

Resistance to treatment with anticancer drugs is a significant obstacle and a fundamental cause of therapeutic failure in cancer therapy. Functional doxorubicin (DOX) nanoparticles for targeted delivery of the classical cytotoxic anticancer drug DOX to tumor cells, using folate-terminated polyrotaxanes along with dequalinium, have been developed and proven to overcome this resistance due to specific molecular features, including a size of approximately 101 nm, a zeta potential of 3.25 mV and drug-loading content of 18%. Compared with free DOX, DOX hydrochloride, DOX nanoparticles, and targeted DOX nanoparticles, the functional DOX nanoparticles exhibited the strongest anticancer efficacy in vitro and in the drug-resistant MCF-7/ Adr (DOX) xenograft tumor model. More specifically, the nanoparticles significantly increased the intracellular uptake of DOX, selectively accumulating in mitochondria and the endoplasmic reticulum after treatment, with release of cytochrome C as a result. Furthermore, the caspase-9 and caspase-3 cascade was activated by the functional DOX nanoparticles through upregulation of the pro-apoptotic proteins Bax and Bid and suppression of the antiapoptotic protein Bcl-2, thereby enhancing apoptosis by acting on the mitochondrial signaling pathways. In conclusion, functional DOX nanoparticles may provide a strategy for increasing the solubility of DOX and overcoming multidrug-resistant cancers.

## INTRODUCTION

The clinical failure of chemotherapy in cancer could be associated with the conventional administration of anticancer drugs as ‘free’ drugs. Such administration leads to a limited drug concentration at tumor sites due to non-targeted distribution throughout the body tissues [1]. Targeted drug delivery offers a potential alternative strategy for chemotherapy.

Another major problem in the clinical treatment of cancer with chemotherapeutic drugs is multidrug resistance (MDR) in tumor cells. MDR involves acquired and intrinsic resistance. Acquired resistance in cancer may derive from a drug stimulus, leading to the overexpression of ATP-binding cassette (ABC) transporters and the subsequent efflux of anticancer drugs from within cancer cells [2]. Intrinsic resistance is mainly associated with mitochondria, which are considered the major powerhouses of cells, playing central roles in energy metabolism and in apoptosis [3].

The induction of cancer cell apoptosis involves several factors, including activation of genes encoding pro-apoptotic proteins and inhibition of genes encoding antiapoptotic proteins, which initiates the apoptosis of cancer cells and the opening of mitochondrial permeability transition pores. These events result in the release of cytochrome C from mitochondria into the cytoplasm and activation of the apoptotic protein enzyme caspase-9, which leads to downstream activation of caspase-3 [4, 5]. A number of mechanisms, such as Bax/Bak or Bcl channels, could cause the release of apoptogenic factors [6, 7].

**Table 1:** 

	Blank nanoparticles	DOX nanoparticles	Targeting DOX nanoparticles	Functional DOX nanoparticles
Particle size(nm)	90±1.80	120±2.40	104±2.50	99±2.52
Zeta potential	−1.65±0.54	−3.56±1.45	−1.68±0.77	3.25±1.55
(mV)				
PDI	0.198±0.003	0.201±0.004	0.224±0. 003	0.243±0. 008
Encapsulation	-	78.45±4.34	80.21±3.43	84.21±3.75
(%)				
DLC(%)	-	17.0±0.6	17.6±0.6	18.0±0.7

Accordingly, mitochondria have been considered as a potential drug delivery target in the treatment of malignant cancers, leading to the design of mitochondria-targeting, anticancer drug-loaded nanoparticles. Mitochondria in cancers are negatively charged, whereas mitochondria-targeting, drug-loaded nanoparticles carry a number of positive charges, thereby enhancing any possible binding between the mitochondria and the nanocarriers internalized by cancer cells [8, 9].

Dequalinium (DQA) is an amphipathic, cationic compound that contains two cationic aminoquinaldinium rings separated by a 10-carbon methylene bridge and that can selectively accumulate in mitochondria, driven by the transmembrane electric potential [10, 11]. Therefore, DQA has been used for the selective delivery of drugs into mitochondria [11].

Polyrotaxanes are necklace-like inclusion complexes formed by the host-guest interaction between cyclodextrins and polymeric chains via threading of the polymeric chains into the cavity of the cyclodextrins [12, 13]. Nanoparticles composed of these regular stacks of cyclodextrins along polymeric chains can be self-assembled by polyrotaxanes. α-Cyclodextrin (α-CD) is a cyclic oligosaccharide composed of six D-glucose units linked by 1,4-α-glucosidic bonds that is widely used in pharmaceutical science due to its excellent biocompatibility. Meanwhile, poly(ethylene glycol) (PEG) not only is regarded as a biodegradable material but is also known to achieve prolonged circulation in the blood. The fabrication of nanoparticles via the crystallization of polyrotaxanes has been reported [14-16], and it is evident that α-CD/PEG polyrotaxane nanoparticles are attractive drug carriers.

In a previous study, we successfully synthesized folate-terminated polyrotaxanes (FPRs) that can condense DNA efficiently and that exhibit excellent gene transfection activity compared with Boc-Tyr-terminated polyrotaxanes (BPRs) [17]. Based on the interaction of folate with the folate receptor on MCF cells, a drug encapsulated in the delivery system can effectively bypass P-gp, target a specific site and induce apoptosis [18-20]. However, whether FPRs, as a carrier, can be applied as a delivery drug system that targets drug-resistant cancer cells is unknown.

**Figure 1:**
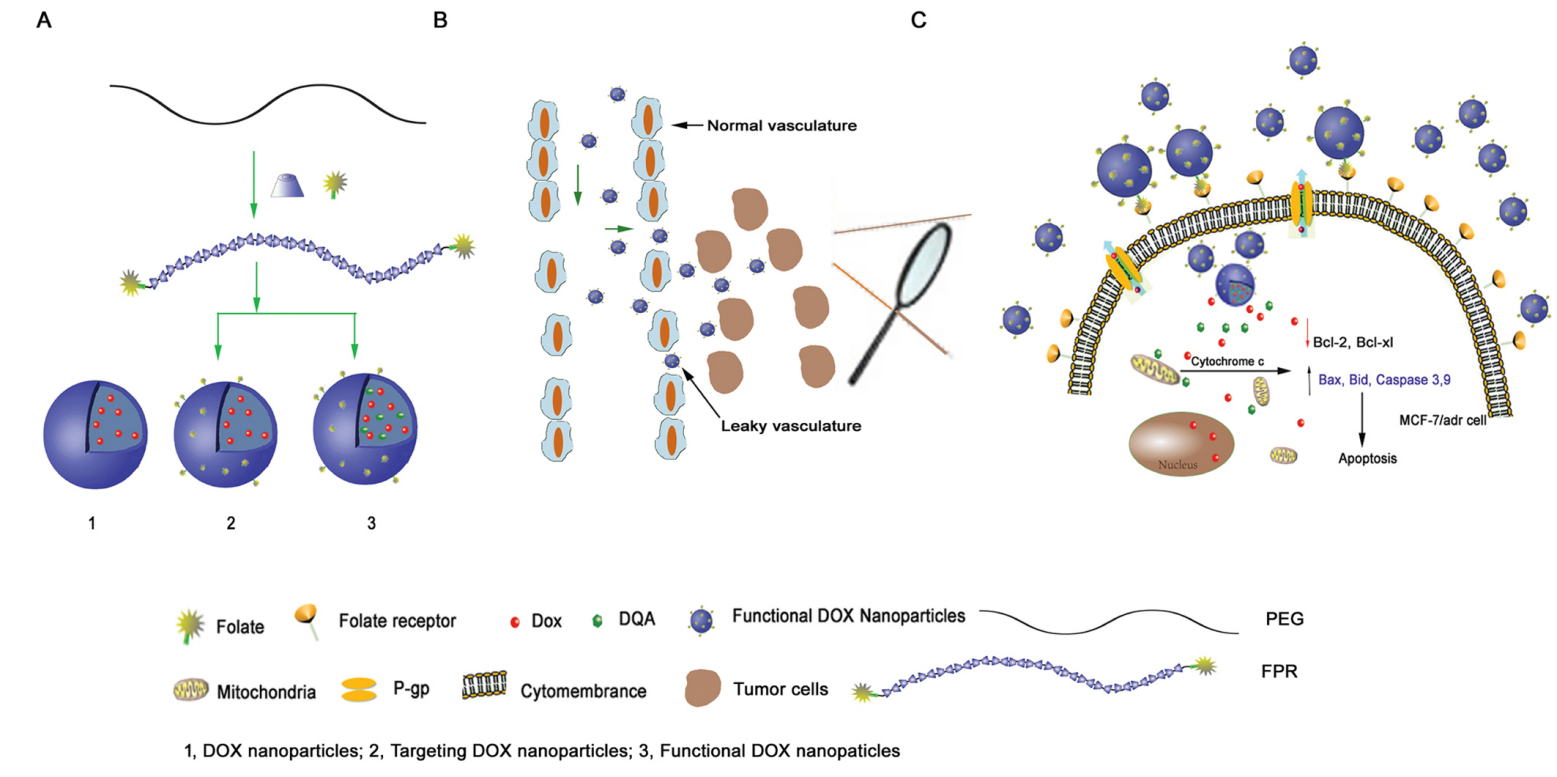
Schematic representations of DOX nanoparticles, targeted DOX nanoparticles and functional DOX nanoparticles (A) and the mechanism of functional DOX nanoparticles' effect on cells with MDR (B, C). The functional DOX nanoparticles, which consisted of FPRs, DQA, and DOX, overcame DOX resistance *in vitro* by targeting the mitochondria of MCF-7/Adr cells.

Doxorubicin (DOX), an anthracycline antibiotic, is a commonly used cytotoxic drug that exhibits a distinct anticancer effect on various human cancers in the clinic. However, the anticancer activity of DOX is significantly limited due to its poor aqueous solubility [21] and due to drug resistance [22]. In the current study, we designed a type of functional DOX nanoparticle that was prepared using FPRs and DQA as nanocarrier-forming materials. The objective of this study was to characterize the functional DOX nanoparticles and their targeting effect, mechanism of action, and anticancer efficacy in resistant tumors (Fig. 1).

## RESULTS

### Characterization of nanoparticles

**Figure 2:**
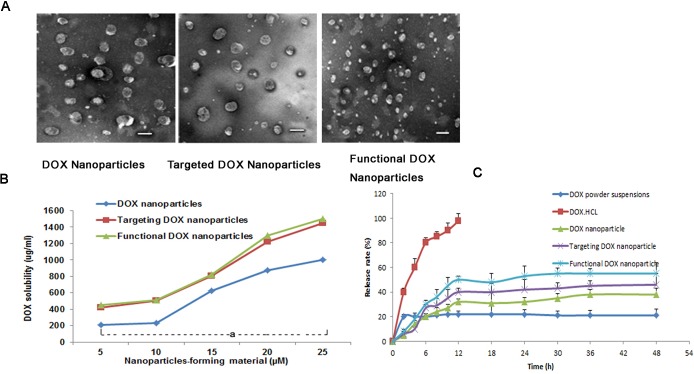
DOX nanoparticles and characterization (A) TEM image of different types of nanoparticles, scale bar = 150 nm. (B) Effect of the nanoparticle-forming material's total concentration on the solubilized DOX concentration in the nanoparticles. ^a^*P*<0.05, compared with DOX nanoparticles. (C) DOX release rates (%) of five different formulations of DOX in PBS containing 10% FBS at 37°C (pH 7.4). The data are presented as the mean ± SD (n=3).

The self-assembly method appears to be particularly suitable for the incorporation of DOX and DQA into BPR- or FPR-based nanoparticles; in the present study, DOX nanoparticles, targeted DOX nanoparticles, and functional DOX nanoparticles were formed. Table 1 lists the average particle sizes, zeta potentials, PDIs, DLCs, and EEs of the various types of nanoparticles (blank nanoparticles, DOX nanoparticles, targeted DOX nanoparticles, and functional DOX nanoparticles). The corresponding average particle sizes were 90±1.80 nm, 120±2.40 nm, 104±2.50 nm, and 99±2.52 nm, respectively. Accordingly, the zeta potential values were −1.65±0.54, −3.56±1.45, −1.68±0.77, and 3.25±1.55, respectively, and the DLC values were 0%, 17.0±0.6%, 17.6±0.6%, and 18.0±0.7%, respectively. For all nanoparticles prepared, the EE of DOX was >80%. For the functional DOX nanoparticles, the PDI values for all micelles were approximately 0.2. Fig. 2A shows the TEM images of the three nanoparticle types after staining with 1% uranyl acetate. Spherical particles of uniform size were observed by TEM, with sizes in the range of 100-120 nm. Fig. 2B demonstrates the effect of the nanoparticle-forming material's total concentration on the solubility of DOX. The results show that the solubility of DOX increased with an increase in the nanoparticle-forming material's total concentration. When the nanoparticle-forming material's concentration reached 25 μM, the solubility values of DOX in the DOX nanoparticles, targeted DOX nanoparticles, and functional DOX nanoparticles were 974±32 μg/ml, 1334±53 μg/ml, 1445±23 μg/ml, respectively.

Fig. 2C shows the rates of *in vitro* DOX release from the drug-loaded nanoparticles in PBS containing 10% FBS. The results show that the rates of DOX release from all nanoparticles were less than 20% at 2 h. Over 12 h, the release rate of DOX·HCL injection was greater than 98% in body fluids, but the rate was less than 10% for DOX powder suspensions. Meanwhile, the rates of release from the DOX nanoparticles, targeted DOX nanoparticles, and functional DOX nanoparticles at 12 h were as follows: 36.0±0.9%, 45.0±1.0% and 50.3±2.4%, respectively. In contrast to the release profile of DOX injection, the profiles of the various types of DOX nanoparticles exhibited delayed release characteristics; after 12 h, the release curves of all DOX-loaded nanoparticles exhibited plateau-like profiles.

### Inhibitory effect on resistant breast cancer cells *in vitro*

**Figure 3:**
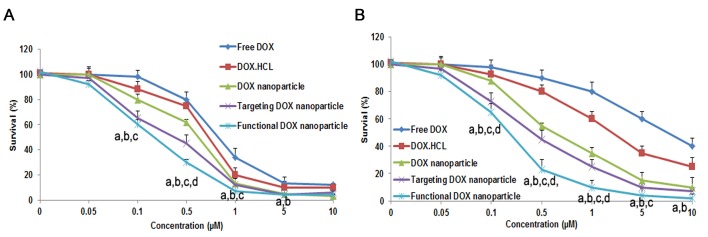
Inhibitory effect of five DOX-containing formulations on the proliferation of MCF-7 cells (A) and MCF-7/Adr cells (B) *in vitro*. The data are presented as the mean ± SD (n=3). Notes: ^a^*P*<0.05, compared with free DOX; ^b^*P*<0.05, compared with DOX·HCL; ^c^*P*<0.05, compared with DOX nanoparticles; ^d^*P*<0.05, compared with targeted DOX nanoparticles.

Fig. 3 shows the inhibitory effect of the different DOX formulations on MCF-7 (Fig. 3A) and MCF-7/Adr (Fig. 3B) cells. When inhibiting MCF-7 cells, the IC50 values of free DOX, DOX·HCL injection, DOX nanoparticles, targeted DOX nanoparticles, and functional DOX nanoparticles were 0.77 μM, 0.68 μM, 0.55 μM, 0.42 μM, and 0.24 μM, respectively. In contrast, the IC50 values of free DOX, DOX injection, DOX nanoparticles, targeted DOX nanoparticles, and functional DOX nanoparticles were 7.22 μM, 6.54 μM, 0.72 μM, 0.46 μM, and 0.30 μM, respectively, when inhibiting MCF-7/Adr cells.

### Cellular uptake and mitochondrial targeting

### Cellular uptake

**Figure 4:**
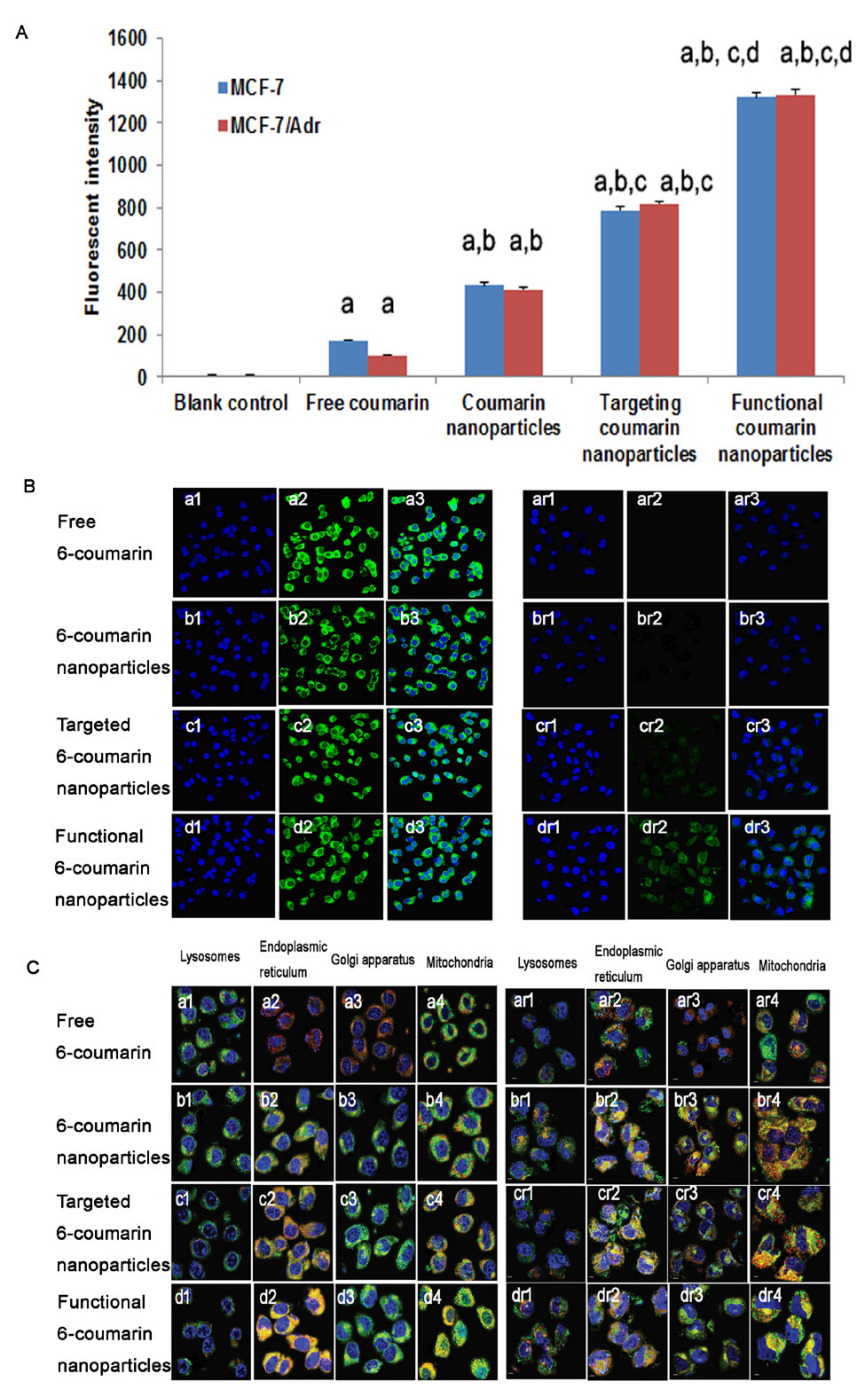
Uptake after incubation with varying formulations Uptake of drugs by MCF-7 cells and MCF-7/Adr cells (A). The data are presented as the mean ± SD (n=3). Notes: ^a^*P*<0.05, compared with blank control; ^b^*P*<0.05, compared with free 6-coumarin; ^c^*P*<0.05, compared with 6-coumarin nanoparticles;^d^*P*<0.05, compared with targeted 6-coumarin nanoparticles. Uptake of different rhodamine 123-containing formulations by MCF-7 cells (B, left) and MCF-7/Adr cells (B, right). Notes: The blue color denotes the nuclei of MCF-7 or MCF-7/Adr cells stained with Hoechst 33342. The green color denotes rhodamine 123. The overlapping images show both Hoechst 33342 and rhodamine 123 in MCF-7 or MCF-7/Adr cells. (C) Intracellular accumulation in several cytoplasmic organelles in MCF-7 (C, left) and MCF-7/Adr (C, right) cells after treatment with free 6-coumarin, 6-coumarin nanoparticles, targeted 6-coumarin nanoparticles, or functional 6-coumarin nanoparticles. Notes: The blue and green colors denote Hoechst 33342 and 6-coumarin, respectively. The red color denotes lysosomes, the ER, the GA, and mitochondria stained with LysoTracker Red DND-99, ER-Tracker Red, BODIPY TR ceramide complexed to BSA, or MitoTracker Deep Red 633, respectively. The yellow color (merged image) indicates 6-coumarin in different organelles.

Fig. 4A presents flow cytometry images of the cellular uptake of the various DOX formulations. After applying blank medium, free 6-coumarin, 6-coumarin nanoparticles, targeted 6-coumarin nanoparticles, or functional 6-coumarin nanoparticles, the geometric mean fluorescence intensities in MCF-7 cells were 5.93±1.12, 213.48±2.36, 450.34±12.22, 787.55±10.2 and 1322.27±32.12 (Fig. 4A, blue), respectively, and those in MCF-7/Adr cells were 6.54±1.21, 111.23±3.46, 428.43±12.21, 820.34±23.13 and 1335.67±52.32 (Fig. 4A, red), respectively. The geometric mean fluorescence intensities indicated the uptake of 6-coumarin into the cells.

MCF-7 and MCF-7/Adr cells were observed by confocal microscopy at 2 h after applying free rhodamine 123, rhodamine 123 nanoparticles, targeted rhodamine 123 nanoparticles or functional rhodamine 123 nanoparticles, as shown in Fig. 4B (left and right, respectively). Distinct cellular accumulation resulted from the fact that the highly hydrophilic free rhodamine 123 readily diffused into the MCF-7 cells. Compared with those of the rhodamine 123 nanoparticles and targeted rhodamine 123 nanoparticles, the overlaid images of the functional rhodamine 123 nanoparticles displayed more intense green fluorescence for rhodamine 123 in the cell cytoplasm. In contrast, the resistant MCF-7/Adr cells showed only limited fluorescence intensity for free rhodamine 123, and the overlaid images of the functional rhodamine 123 nanoparticles displayed the strongest fluorescence intensity in the cell cytoplasm compared with the intensities demonstrated by the rhodamine 123 nanoparticles and the targeted rhodamine 123 nanoparticles. Interestingly, the fluorescence of rhodamine 123 in functional DOX nanoparticle-treated MCF-7/Adr cells was nearly the same as that in functional DOX nanoparticle-treated MCF-7 cells, indicating a loss of DOX resistance in the MCF-7/Adr cells.

### Subcellular localization in resistant breast cancer cells

Confocal fluorescence images of MCF-7 cells (Fig. 4C, left) and MCF-7/Adr cells (Fig. 4C, right) incubated with free 6-coumarin, 6-coumarin nanoparticles, targeted 6-coumarin nanoparticles, or functional 6-coumarin nanoparticles are shown in Fig. 4. Yellow indicates colocalization of the nanoparticles with organelle-selective dyes, and red indicates non-colocalization. The red fluorescence of the organelle-selective dyes was observed in the presence of free 6-coumarin, indicating that free 6-coumarin did not localize in lysosomes, the ER, the GA or mitochondria (Fig. 4C, a1-a4, ar1-ar4). Furthermore, the patterns of yellow fluorescence indicated that the 6-coumarin nanoparticles, targeted 6-coumarin nanoparticles and functional 6-coumarin nanoparticles did not accumulate in lysosomes (Fig. 4C, b1-d1, br1-dr1) but specifically accumulated in the ER (Fig. 4C, b2-d2, br2-dr2) and GA (Fig. 4C, b3-d3, br3-dr3). Additionally, the functional 6-coumarin nanoparticles profoundly and selectively accumulated in mitochondria (Fig. 4C, d4, dr4) relative to accumulation in the ER and GA.

### Drug content in the mitochondrial fraction

**Figure 5:**
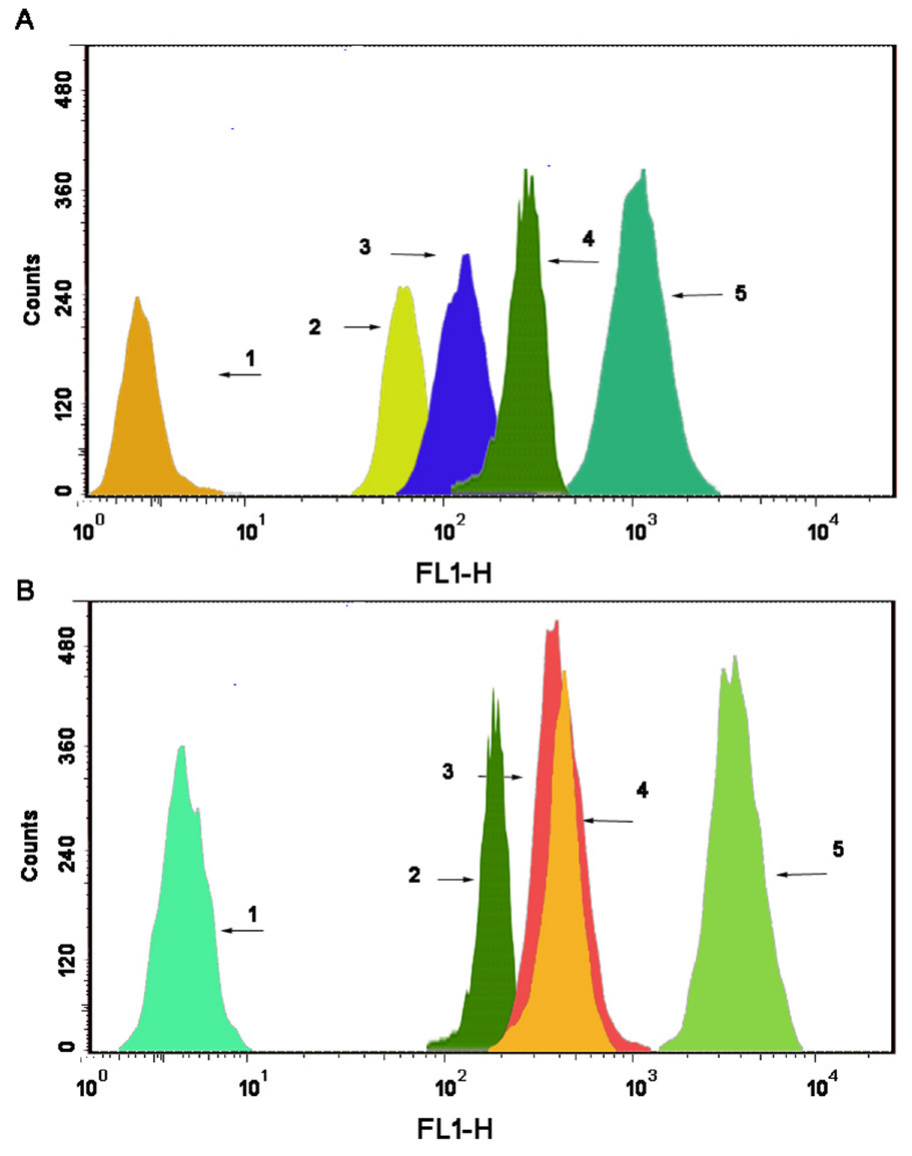
Drug content of the mitochondrial fraction in MCF-7 cells (A) and MCF-7/Adr cells (B) after applying different formulations, as determined by flow cytometry. The abscissa indicates the fluorescence intensity of a 6-coumarin formulation internalized by the cancer cells, and the ordinate represents the cell counts. Notes: A1 and B1, blank control; A2 and B2, free 6-coumarin; A3 and B3, 6-coumarin nanoparticles; A4 and B4, targeted 6-coumarin nanoparticles; A5 and B5, functional 6-coumarin nanoparticles.

As shown in Fig. 5, the 6-coumarin content in single mitochondria was observed after the addition of blank culture medium, free 6-coumarin, 6-coumarin nanoparticles, targeted 6-coumarin nanoparticles or functional 6-coumarin nanoparticles to MCF-7 (Fig. 5A) or MCF-7/Adr (Fig. 5B) cells for 6 h. Compared with the intensities observed after applying the free 6-coumarin, 6-coumarin nanoparticles, or targeted 6-coumarin nanoparticles, the fluorescence intensity of the mitochondria after applying the functional 6-coumarin nanoparticles was 26.21-fold, 16.43-fold and 6.42-fold higher, respectively. In contrast, the fluorescence intensity of the functional 6-coumarin nanoparticles was approximately 9.54-fold higher than that of free 6-coumarin, 7.52-fold higher than that of the 6-coumarin nanoparticles, and 6.83-fold higher than that of the targeted 6-coumarin nanoparticles in the mitochondria of the MCF-7/Adr cells.

### Release of cytochrome C from mitochondria

**Figure 6:**
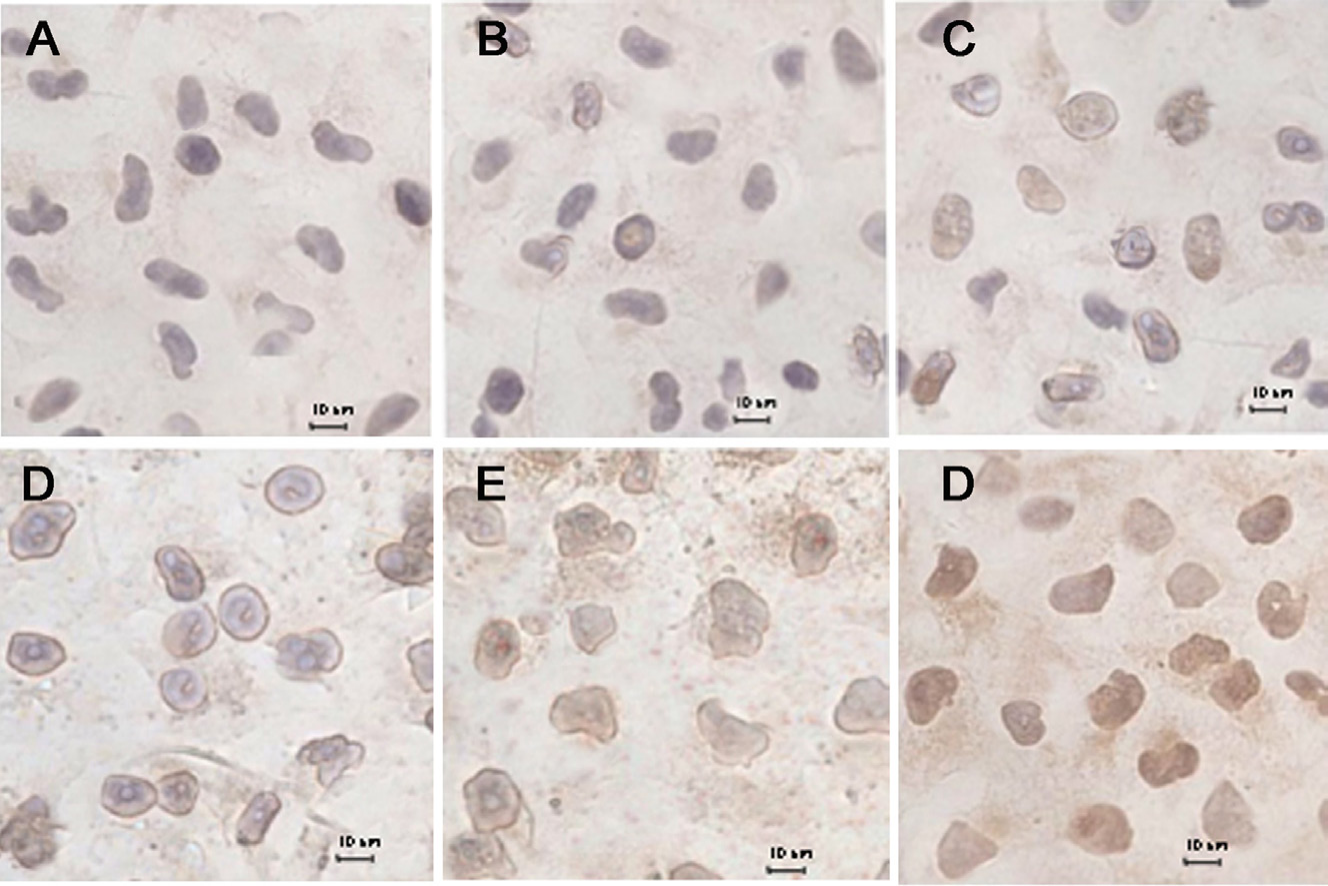
Immunohistochemical staining of cytochrome C translocated from mitochondria to the cytosol in MCF-7/Adr cells after incubation with different formulations, including a blank control (A), free DOX (B), DOX·HCL (C), DOX nanoparticles (D), targeted DOX nanoparticles (E), and functional DOX nanoparticles (F).

Fig. 6 shows the release of cytochrome C from the mitochondria of MCF-7/Adr cells after the addition of blank culture medium, free DOX, DOX·HCL, DOX nanoparticles, targeted DOX nanoparticles, or functional DOX nanoparticles. The results show that little release occurred in the blank-control group (Fig. 6A) and that slight release occurred in response to the free DOX (Fig. 6B), DOX·HCL (Fig. 6C), DOX nanoparticles (Fig. 6D) and targeted DOX nanoparticles (Fig. 6E). In contrast, a large amount of cytochrome C release from mitochondria (Fig. 6F) was observed in response to the functional DOX nanoparticles, as indicated by brown staining, demonstrating that the nanoparticles entered the mitochondria.

### *In vitro* apoptosis-inducing effect

**Figure 7:**
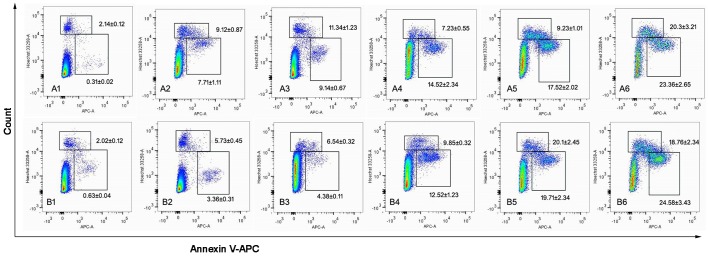
The cell apoptosis rate was detected by flow cytometry MCF-7 cells (A) and MCF-7/Adr cells (B) were treated with different formulations each containing a total DOX concentration of 2 μM for 24 h. Notes: A1 and B1, control (PBS); A2 and B2, DOX solution; A3 and B3, DOX·HCL; A4 and B4, DOX nanoparticles; A5 and B5, targeted DOX nanoparticles; A6 and B6, functional DOX nanoparticles.

Fig. 7 depicts apoptosis-inducing effects in MCF-7 (Fig. 7A) and MCF-7/Adr (Fig. 7B) cells after applying blank culture medium, free DOX, DOX nanoparticles, targeted DOX nanoparticles, or functional DOX nanoparticles *in vitro*. The apoptosis-inducing effects were evaluated by calculating the apoptotic percentage during the early period. After applying the blank culture medium, free DOX, DOX-loaded nanoparticles, targeted DOX nanoparticles, or functional DOX nanoparticles, the induced apoptotic percentages in MCF-7 cells were 7.17%, 9.14%, 14.52%, 17.52%, and 23.2%, respectively, and those in MCF-7/Adr cells were 3.36%, 4.38%, 12.5%, 19.7%, and 24.5%, respectively.

### Apoptosis signaling pathways

### Caspase activities

**Figure 8:**
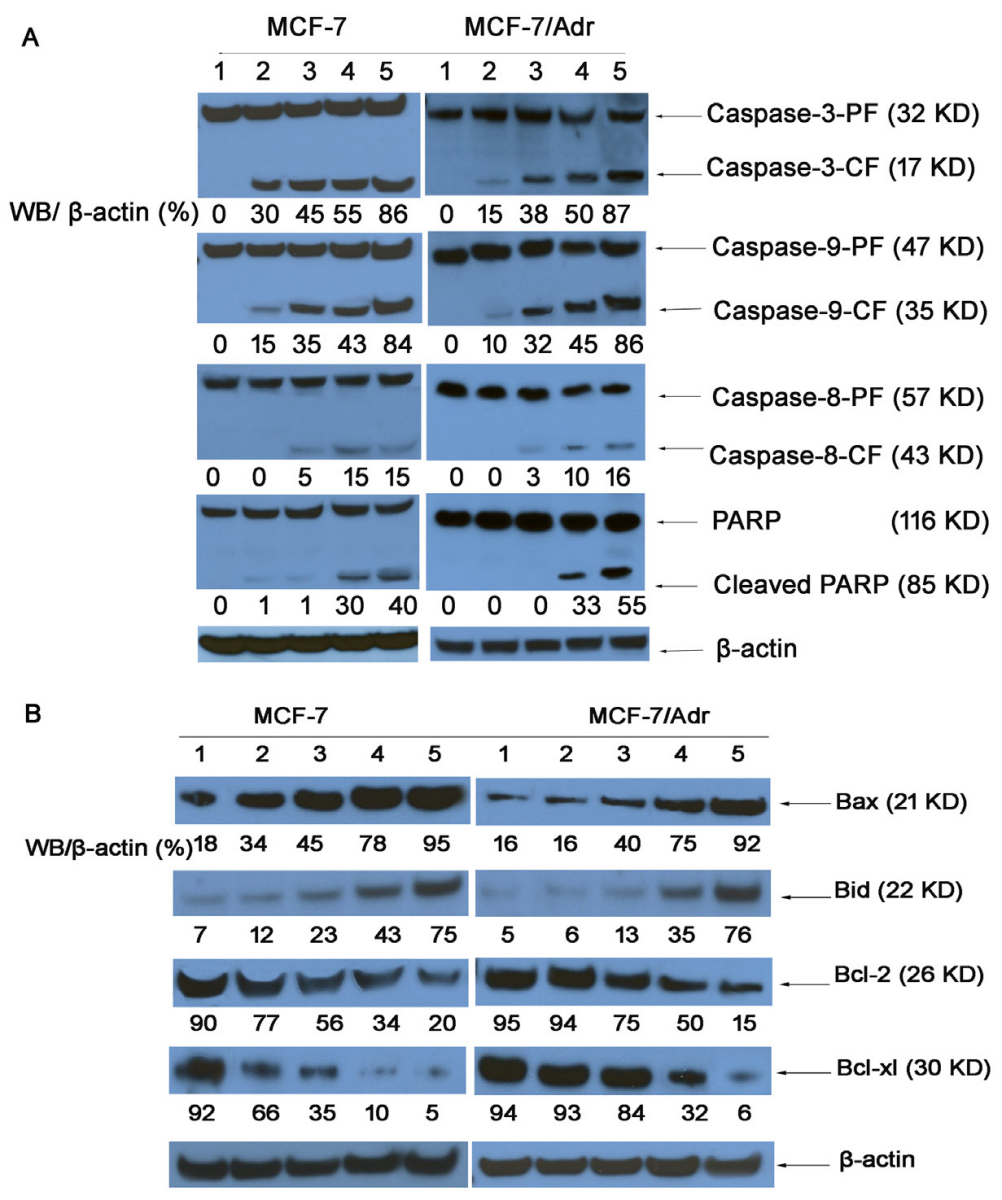
Expression of proteins involved in the apoptosis signaling pathways in MCF-7 and MCF-7/Adr cells, as determined by western blotting (1) Control (PBS); (2) free DOX; (3) DOX·HCL; (4) DOX nanoparticles; (5) targeted DOX nanoparticles; and (6) functional DOX nanoparticles. Activity ratios of caspase-3 and caspase-9 (A) and expression ratios of the pro-apoptotic proteins Bax and Bid and the anti-apoptotic proteins Bcl-2 and Bcl-xl (B) in MCF-7 and MCF-7/Adr cells after incubation with varying formulations. β-actin was also assessed by western blotting. All protein levels were quantified densitometrically and normalized to β-actin.

Western blotting revealed the activities of a series of caspases and of PARP in MCF-7 and MCF-7/Adr cells (Fig. 8A). The expression of caspase-9 and caspase-3 in MCF-7 and MCF-7/Adr cells treated with various nanoparticle formulations was significantly higher than the expression of caspase-8, indicating that caspase-9 and caspase-3 were activated. Interestingly, the caspase-9 activity ratios in MCF-7/(MCF-7/Adr) cells were 15%/10%, 35%/32%, 43%/45%, and 84%/86%, respectively. In contrast, the caspase-3 activity ratios in MCF-7/(MCF-7/Adr) cells were 30%/15%, 45%/38%, 55%/50%, and 86%/87%, respectively, indicating that drug resistance was overcome.

### Bcl-2 family protein expression

Fig. 8B illustrates the activities of pro-apoptotic proteins (Bax and Bid) and anti-apoptotic proteins (Bcl-2 and Bcl-xl) in MCF-7 and MCF-7/Adr cells, as determined by western blotting. The Bax activity ratios for functional DOX nanoparticles in the MCF-7/(MCF-7/Adr) cells were 5.27/5.75-fold higher than for the blank control, 2.79/5.75-fold higher than for free DOX, 2.11/2.30-fold higher than for DOX nanoparticles, and 1.22/1.22-fold higher than for targeted DOX nanoparticles. Additionally, the Bid activity ratios for functional DOX nanoparticles in the MCF-7/(MCF-7/Adr) cells were 10.71/14.2-fold higher than for the blank control, 6.25/12.66-fold higher than for free DOX, 3.26/5.84-fold higher than for DOX nanoparticles, and 1.74/2.17-fold higher than for targeted DOX nanoparticles. However, the Bcl-2 activity ratios for functional DOX nanoparticles, targeted DOX nanoparticles, DOX nanoparticles, and free DOX in the MCF-7/(MCF-7/Adr) cells were 5%/6%, 10%/32%, 35%/84%, 66%/93%, and 92%/94%, respectively, suggesting that the functional DOX nanoparticles can inhibit resistant MCF-7 cells.

### Anticancer efficacy in resistant human breast cancer xenografts

**Figure 9:**
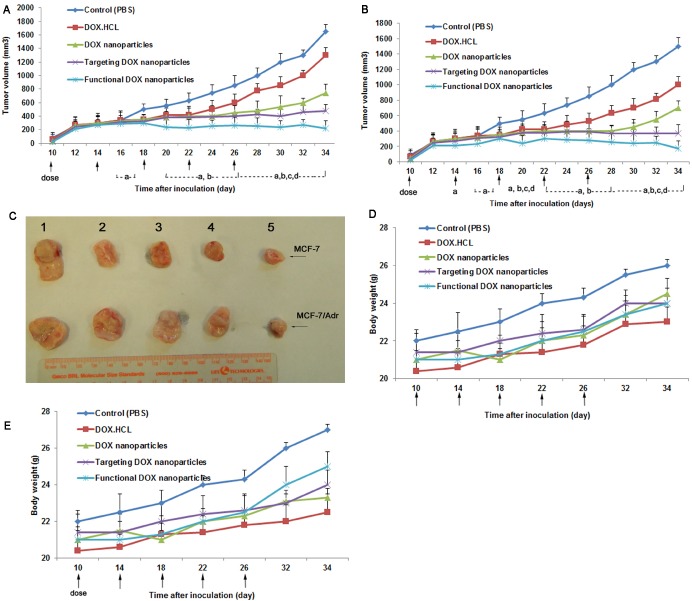
Anticancer efficacy of functional DOX nanoparticles in MCF-7- or MCF-7/Adr-bearing Balb/c mice Mice were injected intravenously with DOX·HCL, DOX nanoparticles, targeted DOX nanoparticles, or functional DOX nanoparticles at doses equivalent to 5 mg DOX per kg on days 10, 14, 18, 22, and 26 (PBS was used as a control). The changes in (A, B) tumor volume and (D, E) body weight in the MCF-7- or MCF-7/Adr-bearing Balb/c mice after administration are shown. (C) After 34 days, the tumors were excised from the MCF-7- or MCF-7/Adr-bearing mice in each group. The data are presented as the mean ± SD (n=5). ^a^*P*<0.05, compared with control; ^b^*P*<0.05, compared with DOX·HCL; ^c^*P*<0.05, compared with DOX nanoparticles; ^d^*P*<0.05, compared with targeted DOX nanoparticles.

Fig. 9 depicts anticancer efficacy in the MCF-7 and MCF-7/Adr xenograft model (Fig. 9C). The results show that DOX·HCL, he DOX nanoparticles, and the targeted DOX nanoparticles had a minimal inhibitory effect on the MCF-7 (Fig. 9A) and MCF-7/Adr (Fig. 9B) xenografts when administered by intravenous injection (30.2±14.3%/18.2±12.34%, 52.2±10.12%/58.8±15.5%, and 75.6±15.25%/78.23±24.5%, respectively, at day 34). In contrast, the functional DOX nanoparticles had the best antitumor efficacy when administered by intravenous injection, as reflected by the lower tumor volume (86.4±10.8%/88±21.12% at day 34).

Body weight changes in the tumor-bearing mice were observed during the study of antitumor efficacy in MCF-7 (Fig. 9D) and MCF-7/Adr (Fig. 9E) xenografts. Given the results observed for both administration types, significant body weight loss was not observed in the mice after administration of functional DOX nanoparticles but was observed in the mice given DOX·HCL.

## DISCUSSION

MDR is a main reason for poor prognosis in chemotherapy. MDR in cancer may be caused by many factors, including cancer cell membrane-related drug resistance due to overexpression of ABC transporters and mitochondria-related drug resistance, which are involved in overall resistance to chemotherapy. However, the specific delivery of an anticancer drug to a tumor site can significantly improve the efficacy of chemotherapy [28]. Thus, the synthesis of targeted nanoparticles is a promising approach for the targeted delivery of anticancer drugs.

In the present study, functional DOX nanoparticles were developed using FPRs and DQA as the nanoparticle-forming materials. The constructed functional DOX nanoparticles had the following physicochemical features: a small particle size (approximately 100 nm), a slightly positive potential, high EE (85%), high DLC (>18%), high DOX solubility (>1 mg/ml) (Table 1), and delayed drug release (Fig. 2A). As shown in Table 1, the size of the drug-loaded nanoparticles was larger than that of blank nanoparticles due to the incorporation of the drug into the nanoparticles' matrix, which increased the concentration of the solid phase and reduced the zeta potential of the prepared nanoparticles. However, the folate-modified nanoparticles were relatively smaller than unmodified nanoparticles; this disparity may have been caused by the enhanced hydrophilicity of the nanoparticles' surface due to the incorporation of folate. The zeta potentials of empty nanoparticles, DOX nanoparticles, and targeted DOX nanoparticles were estimated to range between −1 mV and −3.5 mV. Interestingly, the functional DOX nanoparticles instead displayed positive zeta potential values (3.5 mV). The reason for this different result may have been the addition of DQA. However, the main problem faced by nanodrug carriers is low DLC; in most polymeric drug nanocarriers, such as nanospheres and micelles, the DLC is less than 10% [19, 29]. Yang et al. reported that a low dose of anticancer drugs is less effective against drug-resistant cancers [30]. For all three polyrotaxane nanoparticle formulations presented here, however, the DLC and EE were greater than 17% and 85%, respectively. This explains why polyrotaxane is superior to other carriers for drug delivery. Furthermore, their small particle size and high EE enable functional DOX nanoparticles to be transported into tumor tissue and allow for greater accumulation of the nanoparticles in tumor tissue because of the enhanced permeability retention (EPR) effect [31]. The improved solubility of DOX due to the lipophilic portions of α-CD and PEG in functional nanoparticles makes it possible to avoid the use of the anaphylactic excipient Cremophor EL. Lower release during the initial 2 h (<30%) would be beneficial for preventing rapid leakage during the delivery and blood/lymphatic circulation of drug-loaded nanoparticles, thereby possibly increasing the accumulation of the drug in tumor masses.

In the cytotoxicity assay in the present study, the functional DOX nanoparticles exhibited the strongest inhibitory effect on the breast cancer cells, and particularly the resistant MCF-7/Adr cells (Fig. 3B). The mechanism of the enhanced inhibitory effect could involve the following factors: punctuate mitochondrial colocalization (Fig. 4A) and higher mitochondrial uptake (Fig. 4B). Regarding intracellular uptake by resistant MCF-7/Adr cells, the extent of uptake was ranked as follows: functional DOX nanoparticles > targeted DOX nanoparticles > DOX nanoparticles > DOX·HCL > free DOX. These results are related to the folate and DQA added as nanoparticle-forming materials. It has been reported that a dosage formulated with folate can bypass P-gp function in resistant cells and to inhibit cancer cells [32, 33]. Moreover, in the current study, the increased solubility of DOX in the functional nanoparticles enhanced their intracellular uptake and resulted in decreased DOX efflux. DQA, as a mitochondria-tropic molecule with a delocalized charge center, has been used to determine the membrane potential of mitochondria and has been applied to deliver DNA to the mitochondria of living cells [11]; DQA can also further promote uptake by cancer cells via the electrostatic interaction between positively charged functional nanoparticles and the negatively charged sites on the tumor cell surface. Consequently, functional DOX nanoparticles that contain DQA have a stronger inhibitory effect on resistant MCF-7/Adr cells.

Selective drug delivery to subcellular organelles requires an understanding of the cellular distribution of nanoparticles. In the present study, laser confocal microscopy analysis of live cells revealed the localization of nanoparticles in the ER (Fig. 4C, b2-d2, br2-dr2) and GA (Fig. 4C, b3-d3, br3-dr3) by triple labeling, suggesting that the nanoparticles were transported to the two suborganelles after being endocytosed intact by the cells. In contrast, the fluorescence of LysoTracker (red) was distinct from the fluorescence of 6-coumarin-labeled nanoparticles (green) (Fig. 4C, b1-d1, br1-dr1), indicating that the nanoparticles did not localize in lysosomes. This finding could be not attributed to the lysosomes' distribution of small nanoparticle in functional nanoparticles.

Due to effect of the DQA in the functional nanoparticles, the functional 6-coumarin nanoparticles specifically accumulated in mitochondria, whereas the 6-coumarin nanoparticles and targeted 6-coumarin nanoparticles did not significantly accumulate in mitochondria (Fig. 4C, d4, dr4). The mitochondrial fraction uptake assay (Fig. 5A and B) further demonstrated the mitochondrial targeting effect of the nanoparticles. Specifically, the intrinsic resistance of the cancer cells to DOX was overcome by the specific localization of the functional nanoparticles in mitochondria. As mitochondria-targeting DOX nanocarriers result in robust anticancer efficacy in xenografted resistant breast cancers in mice [34], the localization of functional DOX nanoparticles in mitochondria and the ER should promote and enhance the pro-apoptotic action of DOX, leading to expansion of its anticancer efficacy.

The release of cytochrome C is one of the crucial steps in defining the role of mitochondria during apoptosis. The results obtained from immunohistochemical staining indicate that the functional DOX nanoparticles induced significant release of cytochrome C (Fig. 6). In contrast, not even slight release of cytochrome C was observed for free DOX, DOX·HCL, DOX nanoparticles, or targeted DOX nanoparticles.

Current treatment approaches aim to induce cancer cell apoptosis, a form of programmed cell death that is responsible for tissue homeostasis [35-37]. The intrinsic apoptosis pathway is initiated in mitochondria that are permeabilized in response to intracellular death signals, resulting in the release of cytochrome c as well as other apoptogenic factors into the cytosol, where caspase-9 and caspase-3 are activated [38]. Extrinsic apoptosis signaling is initiated by the binding of death receptor ligands, such as tumor necrosis factor-related apoptosis-inducing ligand (TRAIL) or CD95 ligand, to their cognate death receptors at the cell membrane [39]. Fig. 8 shows that after applying functional DOX nanoparticles, the activities of caspase-3 and caspase-9 were significantly enhanced in both MCF-7 and MCF-7/Adr cells, further indicating the involvement of mitochondrial signaling pathways in subsequent apoptosis. Interestingly, the expression of caspase-3 and caspase-9 in response to functional DOX nanoparticles was nearly equal between MCF-7 and MCF-7/Adr cells, suggesting that P-gp was overcome by the functional DOX nanoparticles.

Mitochondrial apoptosis is controlled by the Bcl-2 family [40]. Therapeutic strategies targeting Bcl-2 represent a promising prospect for treating many types of cancers [41]; increased expression levels of the proapoptotic Bcl-2 family proteins will promote the apoptosis of cancer cells, whereas increased expression levels of the antiapoptotic Bcl-2 family proteins will preserve cell survival [42]. In the current study, enhanced pro-apoptotic protein (Bax and Bid) expression and reduced anti-apoptotic protein (Bcl-2 and Bcl-xl) expression were induced in functional DOX nanoparticle-treated MCF-7 and MCF-7/Adr cells (Fig. 8B). Compared with free DOX, DOX nanoparticles, and targeted DOX nanoparticles, functional DOX nanoparticles showed an outcome effect. These results indicate that the functional DOX nanoparticles could enhance the apoptosis of drug-resistant MCF-7/Adr cells by activating the pro-apoptotic proteins and suppressing the anti-apoptotic proteins in mitochondria.

Their therapeutic efficacy in the resistant MCF-7/Adr-xenografted nude mice demonstrate that the functional DOX nanoparticles exhibited the most significant antitumor activity among all formulations at comparable doses of DOX, and the tumor growth was markedly inhibited (Fig. 9). In the current study, the addition of DQA and folate to the functional DOX nanoparticles enhanced the permeability of and uptake by the resistant tumors, which in turn enhanced cytotoxicity to the drug-resistant cancer cells. Additionally, the apoptosis-inducing effect of the functional DOX nanoparticles enhanced the overall anticancer efficacy in the drug-resistant cancer cells. Furthermore, the functional DOX nanoparticles improved the pharmacokinetic profile of DOX due to the use of pegylated materials [43, 44], which ultimately resulted in higher accumulation in the tumors.

## CONCLUSIONS

In the present study, new functional DOX nanoparticles were successfully developed to overcome the MDR of cancers due to acquired and intrinsic drug resistance. The functional DOX nanoparticles are small and exhibit a high EE for DOX, and they are stable in blood component-containing systems, with minimal leakage. The marked efficacy of the functional DOX nanoparticles was demonstrated both by treating MCF-7 breast cancer cells and resistant MCF-7/Adr cells *in vitro* and by treating MCF-7 and resistant MCF-7/Adr xenografts in nude mice. The functional DOX nanoparticles specifically could induce apoptosis of the drug-resistant breast cancer cells by releasing cytochrome C and initiating a cascade of caspase-9 and caspase-3 reactions. In addition, the functional DOX nanoparticles could cause apoptosis of the drug-resistant breast cancer cells by activating the pro-apoptotic proteins Bax and Bid and suppressing the anti-apoptotic protein Bcl-2. Therefore, functional DOX nanoparticles have the potential to treat drug-resistant breast cancer.

## MATERIALS AND METHODS

### Preparation of functional DOX nanoparticles

Functional DOX nanoparticles were prepared to overcome the drug resistance of breast cancer (Fig. 1A). Briefly, FPRs (synthesized in our laboratory) and DQA (Hangzhou Sanhe Chemicals, Co., Ltd, Hangzhou, China) (1:0.4 molar ratio, 10 mg) were used as nanoparticle-forming materials after being dissolved in 5 ml water and stirred for 5 min. DOX hydrochloride (DOX·HCL; Beijing Zhongshuo Pharmaceutical Technology Development Co., Ltd, Beijing, China) was deprotonated in water, and the pH value was adjusted to 9.6 to obtain DOX. This DOX (2.5 mg) was then dissolved in 1 ml tetrahydrofuran (THF), and the solution was dripped into the polyrotaxane solution and stirred for 24 h. After the THF was volatilized, 5 ml water was added to the mixture. Next, free DOX and the THF were removed using a centrifugal concentrator with a dialysis membrane (MWCO = 3000 Da). This process was repeated three times. After the centrifugation, the solution was freeze dried to obtain functional DOX nanoparticles.

Targeted DOX nanoparticles and non-targeted DOX nanoparticles were prepared as controls. The targeted DOX nanoparticles were prepared using the same procedures as for the functional DOX nanoparticles, excluding the addition of DQA. The non-targeted DOX nanoparticles (drug:nanoparticle materials = 1:45, w/w) were also prepared using the same procedures as for the functional DOX nanoparticles, but the FPRs were replaced with BPRs. 6-Coumarin (Sigma-Aldrich, Guangzhou, China), rhodamine 123 (Sigma-Aldrich, Guangzhou, China) or DiR (Invitrogen, Guangzhou, China) nanoparticles was incorporated as a fluorescent probe and prepared using the same procedures as for the functional DOX nanoparticles.

### Characterization of nanoparticles

The sizes and polydispersity indexes (PDIs) of the DOX nanoparticles, targeted DOX nanoparticles, and functional DOX nanoparticles were measured using a Malvern Zetasizer Nano ZS (Malvern Instruments Ltd, Malvern, UK).

The size, structures and morphology of the various types of nanoparticles, including DOX nanoparticles, targeted DOX nanoparticles, and functional DOX nanoparticles, were evaluated using transmission electron microscopy (TEM; Hitachi H7650, Japan). For this purpose, each sample (0.5 mg/ml) was resuspended in water and mixed by ultrasonication for 30 s, after which one drop of this suspension was placed over a carbon-coated copper TEM grid (150-mesh; Sangerbio, China) and allowed to dry at room temperature (RT). Images were then visualized at 80 kV by TEM.

An evaluation of the encapsulation efficiency (EE) and drug-loading content (DLC) was performed as previously reported [21]. Briefly, all types of nanoparticles were dissolved in 3 ml DMSO to determine their respective DLCs. The DOX concentration was then tested using a UV-VIS spectrophotometer at 485 nm. The DLC was calculated based on the standard curve obtained for DOX in DMSO. The EE and DLC were specifically calculated according to the following formulae:

EE (%) = (DOX weight measured in nanoparticles / DOX weight added) × 100% (1)

DLC (wt%) = (DOX weight measured in nanoparticles / (total weight of nanoparticle materials added ± DOX weight added) × 100% (2)

### *In vitro* drug release

The drug-loaded nanoparticles were dispersed and diluted in phosphate-buffered saline (PBS) solution to yield a final concentration of 2 mg/ml. The diluted solution (0.5 ml) was transferred to dialysis membrane tubes (MWCO = 3000 Da), which were then immersed in a flask containing 25 ml PBS solution (pH 7.4) and shaken at a speed of 100 rev/min at 37°C. A volume of 0.5 ml release medium was sampled at 0, 2, 4, 6, 8, 10, 12, 18, 24, 30, 36, 42, and 48 h, followed by the immediate addition of an equal volume of fresh release medium. The DOX content was measured by UV-VIS spectrophotometry, as described above. The release rate (RR, %) was calculated using the formula RR = (W_i_ / W_total_) / 100%, where W_i_ is the measured amount of DOX in the release medium at the time point and W_total_ is the total amount of DOX in an equal volume of the nanoparticle suspension before performing the release experiment. The experiment was repeated in triplicate.

### *In vitro* cell culture studies

### Cell lines and cell culture

MCF-7/Adr (a multidrug-resistant variant), a human breast carcinoma cell line selected for drug resistance to DOX, and MCF-7 (human breast cancer cells), the parental line, were kindly donated by the Department of Pharmacology, School of Pharmacy, Guangzhou Medical University (Guangdong, China). These two cell lines are folate receptor-overexpressing carcinoma cell lines. In particular, MCF-7/Adr cells overexpress P-gp and exhibit a drug-resistant phenotype *in vitro* and *in vivo* when grown in Balb/c mice. In several studies, MCF-7 cells have been used as drug-sensitive controls. In the present study, the two cell lines were grown in 75 cm^3^ flasks containing folate-free RPMI-1640 medium supplemented with 10% fetal bovine medium (FBS), 100 U/ml penicillin and 100 mg/ml streptomycin and maintained in an incubator with a humidified 5% CO_2_/95% atmosphere at 37°C. Cells grown to confluence were subcultured every other day by trypsinization with 0.25% trypsin-EDTA and dilution (1/3) in fresh growth medium.

### Inhibitory effect on resistant breast cancer cells

To measure the inhibitory effects of the various DOX formulations on human breast cancer cells, MCF-7 or MCF-7/Adr cells were seeded into 96-well culture plates at 5000 cells/well and cultured at 37°C in a humidified atmosphere containing 5% CO_2_ for 24 h. The cells were then exposed to a series of concentrations of free DOX (DMSO ≤1%), DOX·HCL, DOX nanoparticles, targeted DOX nanoparticles, or functional DOX nanoparticles. Blank culture medium was used as a blank control. After further incubation for 48 h, antiproliferative activity was measured using a sulforhodamine B (SRB) staining assay and a microplate reader (Bio-Rad model 680, Bio-Rad Laboratories, Inc., Shanghai, China) for reading the absorbance at a wavelength of 540 nm, as reported previously [23]. The survival rate was calculated using the following formula: Survival % = (A540 nm for the treated cells / A540 nm for the control cells) × 100%, where A540 nm is the absorbance value. The dose-effect curves were also plotted. All of the experiments were performed in triplicate.

### *In vitro* mitochondrial-targeting effects

Uptake by resistant breast cancer cells

Uptake by MCF-7 cells and MCF-7/Adr cells was measured using a FACScan flow cytometer (Becton Dickinson, San Jose, CA, USA) [24]. Briefly, MCF-7 cells or MCF-7/Adr cells were seeded into 6-well culture plates at a density of 4×10^5^ cells/well and cultured for 24 h in an atmosphere containing 5% CO_2_ at 37°C. After 24 h of incubation, the cells were further treated with free 6-coumarin, 6-coumarin nanoparticles or functional 6-coumarin nanoparticles for 0.5 h under the same conditions. The final concentration of 6-coumarin was 1.0 μM. A sample to which blank medium was added was regarded as a control. After incubation, the cells were washed twice with cold PBS to stop cellular uptake, followed by treatment with 0.25% trypsin-EDTA. The amount of cellular uptake was then measured based on the fluorescence intensity detected using a FACScan flow cytometer, with an event number of 1×10^4^. Each assay was repeated in triplicate.

In a qualitative study, MCF-7 or MCF-7/Adr cells were seeded into chambered coverslips for 24 h at 37°C in the presence of 5% CO_2_. After the addition of 10 μM free rhodamine 123, rhodamine 123 nanoparticles, targeted rhodamine 123 nanoparticles, or functional rhodamine 123 nanoparticles, the cells were further cultured for 2 h. The cells were then washed three times with ice-cold PBS after the medium was removed, and the cell nuclei were stained with Hoechst 333342 (10 μM) for 30 min. Finally, the cells were washed twice with PBS and observed with a laser scanning confocal microscope (Leica SP2, Heidelberg, Germany).

### Subcellular localization in resistant breast cancer cells

Subcellular localization in resistant breast cancer cells was measured using a laser scanning confocal microscope, as previously described [25]. The localization of 6-coumarin or various types of 6-coumarin nanoparticles in subcellular organelles was observed by labeling the cells with fluorescent probes specific to organelle markers, such as LysoTracker, MitoTracker, ER-Tracker or a Golgi marker (all from Invitrogen, China). In particular, MCF-7 or MCF-7/Adr cells were seeded into chambered coverslips and cultured for 24 h at 37°C in the presence of 5% CO_2_, followed by the addition of 1.0 μM free 6-coumarin, 6-coumarin nanoparticles, targeted 6-coumarin nanoparticles or functional 6-coumarin nanoparticles. After the cells were further incubated for 4 h, the drug-containing medium was removed, and the cells were washed three times with ice-cold PBS. The cells were then stained with organelle-selective dye (Molecular Probes, Eugene, OR, USA). More specifically, lysosomes, mitochondria, the endoplasmic reticulum (ER) and the Golgi apparatus (GA) were visualized by staining the cells with 50 nM LysoTracker Red DND-99, 200 nM MitoTracker Deep Red FM, 1 μM ER-Tracker Red (BODIPY TR glibenclamide) or 5 μM BODIPY TR ceramide complexed to BSA, respectively, for 30 min. The cells loaded with organelle markers were washed with PBS, and the cell nuclei were stained with Hoechst 333342 (10 μM) for 30 min. The cells were then washed twice with PBS and observed with a laser scanning confocal microscope.

### Drug content in the mitochondrial fraction

The drug content in the isolated mitochondrial fraction was measured using a FACScan flow cytometer. MCF-7 or MCF-7/Adr cells were first cultured and then treated with 6-coumarin, 6-coumarin nanoparticles, targeted 6-coumarin nanoparticles or functional 6-coumarin nanoparticles for 6 h at a final 6-coumarin concentration of 10 μM. Mitochondria were isolated according to the guide accompanying the cell mitochondria isolation kit (Beyotime Institute of Biotechnology, Haimen, China). Briefly, the cells were reacted with mitochondria extraction reagent (provided in the kit) and stirred in a homogenizer. The suspensions were then centrifuged at 600 g for 10 min, after which the supernatants were collected and further centrifuged at 3500 g for 10 min. The mitochondria were collected from the precipitates, and a FACScan flow cytometer (Becton Dickinson FACSCalibur, Mountain View, CA, USA) was used to quantify the drug content in the mitochondrial fraction, with an event number of 1×10^4^.

### Release of cytochrome C

The release of cytochrome C from MCF-7/Adr cells' mitochondria into the cytosol was measured using a streptavidin-peroxidase immunohistochemical kit (Zhongshan Goldenbridge Biotechnology, Co., Ltd, Beijing, China) [26]. Briefly, after incubation for 24 h, MCF-7/Adr cells were exposed to free DOX, DOX nanoparticles, targeted DOX nanoparticles or functional 6-coumarin nanoparticles or fresh medium as a control. The final concentration of DOX was 2 μM. After incubation for another 12 h, the cells were sequentially treated with 3% H_2_O_2_, blocking buffer (provided in the kit), the primary antibody (anti-cytochrome C; Nanjing KeyGen Biotechnology, Co., Ltd, Nanjing, China), an enhanced secondary antibody (provided in the kit) and an enhanced streptavidin-HRP conjugate (provided in the kit). After color development, the release of cytochrome C was observed under a light microscope.

### Caspase activation

MCF-7/Adr cells were cultured for 12 h and then treated with free DOX, DOX nanoparticles, targeted DOX nanoparticles or functional DOX nanoparticles. Controls were performed by adding blank medium. The final concentration of DOX was 2 μM. After 12 h of incubation, the cells were harvested, lysed, and analyzed by western blotting. The following antibodies were used: anti-caspase-8, anti-caspase-9, anti-caspase-3, and anti-PARP (all from Cell Signaling, Beverly, MA, USA) [27].

### *In vitro* apoptosis-inducing effect

Apoptosis rates were measured using a FITC-annexin V staining kit and a FACScan flow cytometer. The procedure was performed according to the guide accompanying the apoptosis detection kit (Biosea Biotechnology Co., Ltd, Beijing, China). Briefly, MCF-7 or MCF-7/Adr cells were seeded into 6-well culture plates at a density of 5×10^5^ cells/well in 2 ml growth medium. After 24 h, the cells were treated with fresh medium (as a blank control), free DOX, targeted DOX nanoparticles or functional DOX nanoparticles for 12 h in an atmosphere containing 5% CO_2_ at 37°C. The concentration of DOX was 2 μM. The cells were then harvested and suspended in the provided binding buffer, and 5 μl annexin V-FITC was added to the cell suspensions. This mixture was incubated at RT in the dark for 15 min, and then 5 μl propidium iodide (PI; provided in the kit) was added. Finally, the cell samples were analyzed using an FC500 flow cytometer (Beckman Coulter) using the FL1 and FL4 channels for annexin V-Alexa Fluor 488 and PI, respectively. Each assay was performed in triplicate.

### Bcl-2 family protein expression

The expression of Bcl-2 family proteins in MCF-7 and MCF-7/Adr cells was measured using a western blotting assay (antibodies against Bcl-2, Bcl-x, Bax, and Bid from Cell Signaling, Beverly, MA, USA). Briefly, MCF-7 and MCF-7/Adr cells were separately cultured in an atmosphere containing 5% CO_2_ at 37°C for 24 h, followed by the addition of free DOX, DOX nanoparticles, targeted DOX nanoparticles or functional DOX nanoparticles for 24 h. The final concentration of DOX was 2 μM. Control experiments were performed by the addition of culture medium alone. Western blot analysis was then performed as described previously [27].

### Anticancer efficacy in resistant human breast cancer xenografts

Female Balb/c nude mice (initially weighing 18-20 g and obtained from Southern Medical University Health Science Center) were used to investigate antitumor efficacy *in vivo*. All animal experiments were performed according to the institutional and National Institutes of Health guidelines for the care and use of research animals. Briefly, approximately 1×10^7^ MCF-7/Adr cells were suspended in 200 μl serum-free RPMI-1640 culture medium and injected subcutaneously into the right flanks of the nude mice [31]. When the tumors reached a volume of 200-230 mm^3^, the mice were randomly divided into five groups (5 animals each). In each group, one formulation (DOX·HCL, DOX nanoparticles, targeted DOX nanoparticles or functional DOX nanoparticles) was injected intravenously via the tail vein, with a dose of 5 mg of DOX per kg of body weight. In total, five injections were performed at 4-day intervals. The mice were then monitored every other day for tumor progression with a caliper and for weight change with a weighing scale. The tumor volume was calculated using the following formula: (width^2^ × length / 2). The rate of inhibition of tumor growth at day 36 was calculated using the formula Rv = 100% - (V_drug_ / V_saline_) × 100%, where V_drug_ is the tumor volume after treatment with the drug and V_saline_ is the tumor volume after treatment with physiological saline.

### Statistical analysis

The data are presented as the mean ± standard deviation (SD). One-way analysis of variance (ANOVA) was used to determine the significance of differences among groups, after which post hoc tests with Bonferroni correction were used for multiple comparisons between individual groups.
